# Decoding Viral Transmission Dynamics in Structured Populations Using a Microfluidic Herd‐Immunity‐on‐a‐Chip Platform

**DOI:** 10.1002/advs.77383

**Published:** 2026-09-03

**Authors:** Jiande Zhang, Narina Jung, Min‐Hyeok Kim, Wanyoung Lim, Zhenzhong Chen, Byung Mook Weon, Sungsu Park

**Affiliations:** ^1^ School of Mechanical Engineering Sungkyunkwan University (SKKU) Suwon South Korea; ^2^ School of Physics Korea Institute for Advanced Study (KIAS) Seoul South Korea; ^3^ Department of Biomedical Engineering Sungkyunkwan University (SKKU) Suwon South Korea; ^4^ School of Advanced Materials Science and Engineering Sungkyunkwan University (SKKU) Suwon South Korea; ^5^ Institute of Quantum Biophysics (iQB) Sungkyunkwan University (SKKU) Suwon South Korea

**Keywords:** Herd‐Immunity‐on‐a‐chip, mathematical modelling, reproduction number, viral transmission

## Abstract

Understanding how population structure shapes viral transmission remains challenging because most in vitro models assume well‐mixed conditions and lack spatial structure. We present a Herd‐Immunity‐on‐a‐Chip (HIC) platform—a 444‐chamber microfluidic network that recreates structured populations—and apply it to human coronavirus 229E infecting MRC‐5 fibroblasts. By varying initial inoculum (*I*_0_), susceptible density (*S*_0_), cell motility, and the fraction of non‐susceptible cells (*U*_0_), we decode how seeding, density, immunity and movement reshape outbreak trajectories. Increasing *S*_0_ or *I*_0_ raised local contact rates, reduced effective intercellular spacing, and increased the reproduction number (*R*_0_), accelerating spread. In contrast, higher *U*_0_ suppressed transmission; with *U*_0_ ≥ 80% of the fixed uninfected population (*S*_0_ + *U*_0_), outbreaks collapsed (herd immunity). Independently, lowering *S*_0_ slowed early spread but did not, alone, prevent eventual transmission. We integrate the data with mathematical modelling of spatial, contact‐structured transmission to quantify changes in *R*_0_ and apparent herd‐immunity thresholds. HIC offers a generalizable, bench‐top framework for outbreak forecasting and intervention testing.

## Graphical abstract

A microfluidic Herd‐Immunity‐on‐a‐Chip experimentally recreates structured contact networks to quantify viral transmission dynamics. By controlling susceptible, infected, and non‐susceptible populations, the platform reveals how contact topology governs the reproduction number, outbreak propagation, herd‐immunity–like behavior, and intervention efficacy—phenomena that remain largely masked in conventional well‐mixed culture systems ‐ and complements mathematical epidemic models.

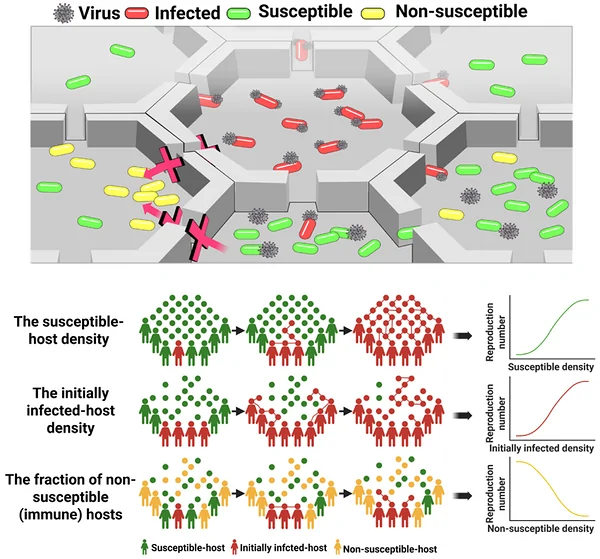

## 1 Introduction

The coronavirus disease 2019 (COVID‐19) pandemic constitutes a major global health crisis, with high morbidity and mortality. Growing evidence links transmission rates to societal factors, including population density, adherence to social distancing, and vaccination coverage. Densely populated regions—such as Hubei Province in China and major U.S. metropolitan areas—exhibited faster spread than less‐populated settings [1], whereas effective distancing measures and vaccination campaigns consistently attenuated transmission [2, 3]. These patterns underscore the interaction between human behavior, public‐health policy, and transmission dynamics, and motivate the development of targeted, evidence‐based interventions.

Traditional compartmental models—such as Susceptible–Infected–Recovered (SIR) [4, 5] and Susceptible‐Exposed‐Infected‐Recovered (SEIR) [6, 7] —have yielded important insights into epidemic dynamics by enabling simulation of epidemiological parameters and evaluation of candidate interventions. However, their standard formulations typically assume homogeneous mixing and time‐invariant transmission rates, assumptions that neglect substantial variability arising from epidemiological, socioeconomic, and behavioral factors [8]. For example, transient surges in effective population density in high‐density settings (e.g., New York City) can amplify contact rates and accelerate spread [9], while early, concentrated outbreaks (e.g., Daegu, Korea) illustrate how the size of the initially infected population shapes subsequent transmission trajectories [10]. Consequently, a gap remains between theoretical predictions from classical models and the context‐dependent realities of transmission in complex human populations.

Conventional cell culture and animal models have advanced our understanding of viral mechanisms but cannot recapitulate the heterogeneous, network‐like interactions that govern transmission in human populations. Simplified biology and interspecies differences limit their translational fidelity, obscuring the roles of contact structure, mobility, and context‐dependent susceptibility in shaping epidemic trajectories [11, 12]. These limitations motivate experimental platforms that capture societal structure and enable quantitative testing of transmission drivers under controlled, reproducible conditions.

**FIGURE 1:**
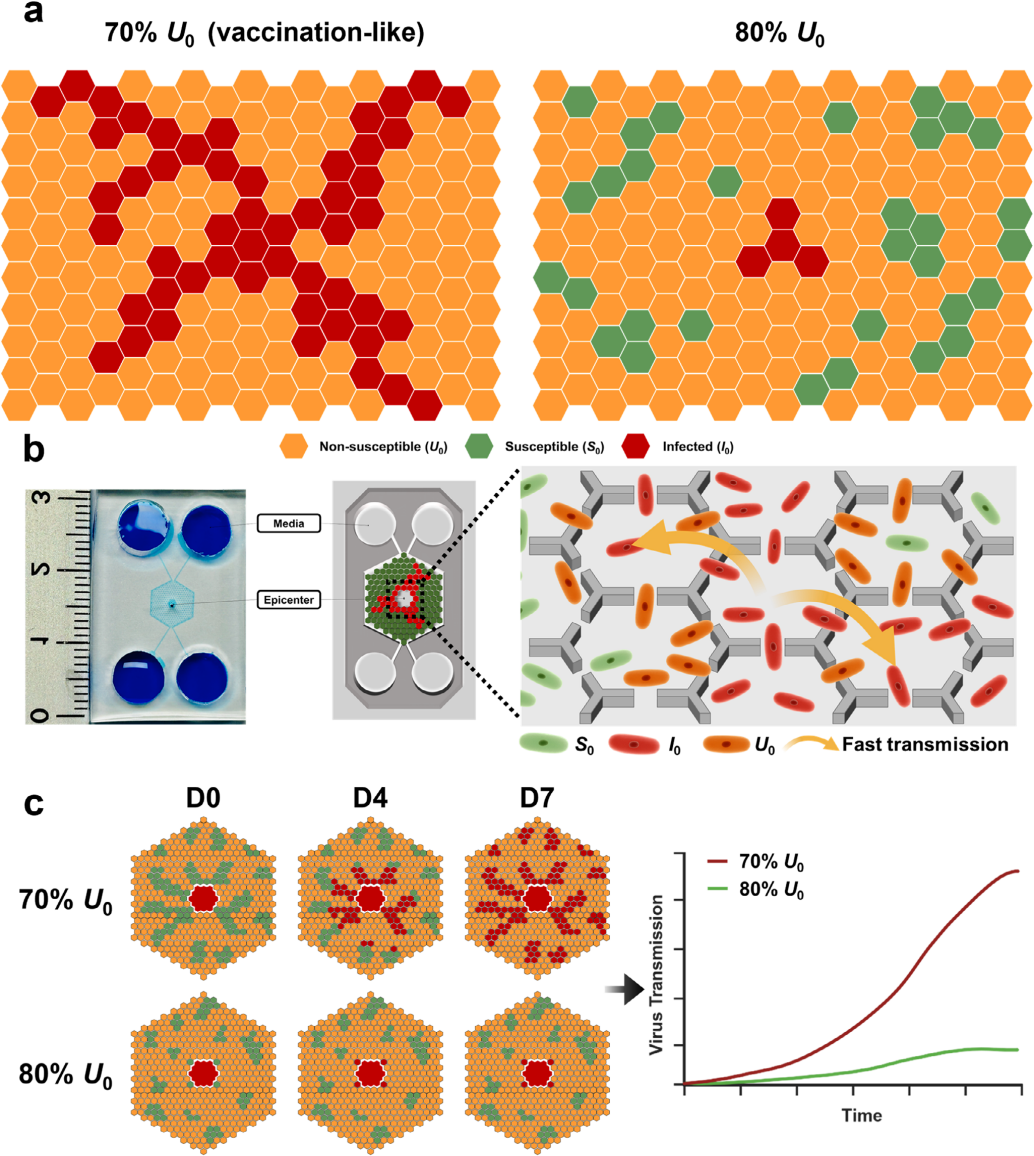
Herd‐Immunity‐on‐a‐Chip (HIC): structured transmission and threshold behavior. (a) Conceptual percolation. In a hexagonal contact lattice, 70% *U*_0_ (yellow) permits infection (red) to percolate through remaining susceptible hosts (green), whereas 80% *U*_0_ fragments connectivity and halts long‐range spread (herd‐immunity–like collapse). (b) Device and relay mechanism. HIC comprises 444 interconnected hexagonal microchambers arranged around a 700 µm‐diameter epicentre (seeding port). Infection relays locally via contacts between initially infected and susceptible cells; *U*_0_ cells provide physical/functional shielding that reduces effective contacts. (c) Readout. Daily snapshots (D0–D7) yield chamber‐resolved trajectories. Enumeration of viable *S* **_0_** provides quantitative transmission metrics (e.g., *R*_0_) and enables detection of a herd‐immunity threshold in the structured network. *U*_0_ represents non‐susceptible cells (APN‐knockdown) used as a phenomenological proxy for vaccination‐induced reductions in susceptibility.

Microfluidic technologies provide rigorously controlled, physiologically relevant microenvironments and have illuminated microbial interaction networks, drug‐resistance evolution, and directed cell migration [13, 14], yet applications to population‐level viral transmission are scarce. We therefore developed Herd‐Immunity‐on‐a‐Chip (HIC), a 444‐chamber hexagonal network with a central epicentre that enforces a structured contact topology, adapting a validated microchamber architecture [15] not previously applied to viral transmission. Conceptually, percolation on this lattice persists at 70% non‐susceptible coverage but fragments at 80%, yielding a herd‐immunity–like collapse (Figure 1a). HIC operationalises this logic by relaying infection locally from the epicentre while non‐susceptible cells reduce effective contacts (Figure 1b). Seven‐day imaging with daily counts produces chamber‐resolved transmission trajectories (Figure 1c). Guided by this framework, we hypothesised that higher *S*_0_ density and larger *I*_0_ accelerate spread, whereas increased *U*_0_ and constrained contacts slow it. We used GFP‐labelled MRC‐5 (*S*_0_), human coronavirus‐229E (HCoV‐229E)–infected lung fibroblast MRC‐5 (*I*_0_), and aminopeptidase N (APN)‐knockdown MRC‐5 (*U*_0_), treating *U*_0_ as a vaccination‐like reduction in susceptibility [16]. Transmission was quantified in both the HIC and 96‐well controls, which respectively realise the structured population and its homogeneous‐mixing limit, allowing the effect of contact topology to be isolated. The reproduction number R_0_ was estimated directly from the experimental S_0_ depletion curves, which were reproduced by a Susceptible–Exposed–Non‐susceptible–Infected‐Dead (SEUID) model. The close experiment–simulation [17] agreement establishes HIC as a quantitative platform to probe transmission drivers and herd‐immunity thresholds.

## 2 Results and Discussion

### 2.1 Susceptible‐Cell Density Governs Transmission in Structured Networks

**FIGURE 2:**
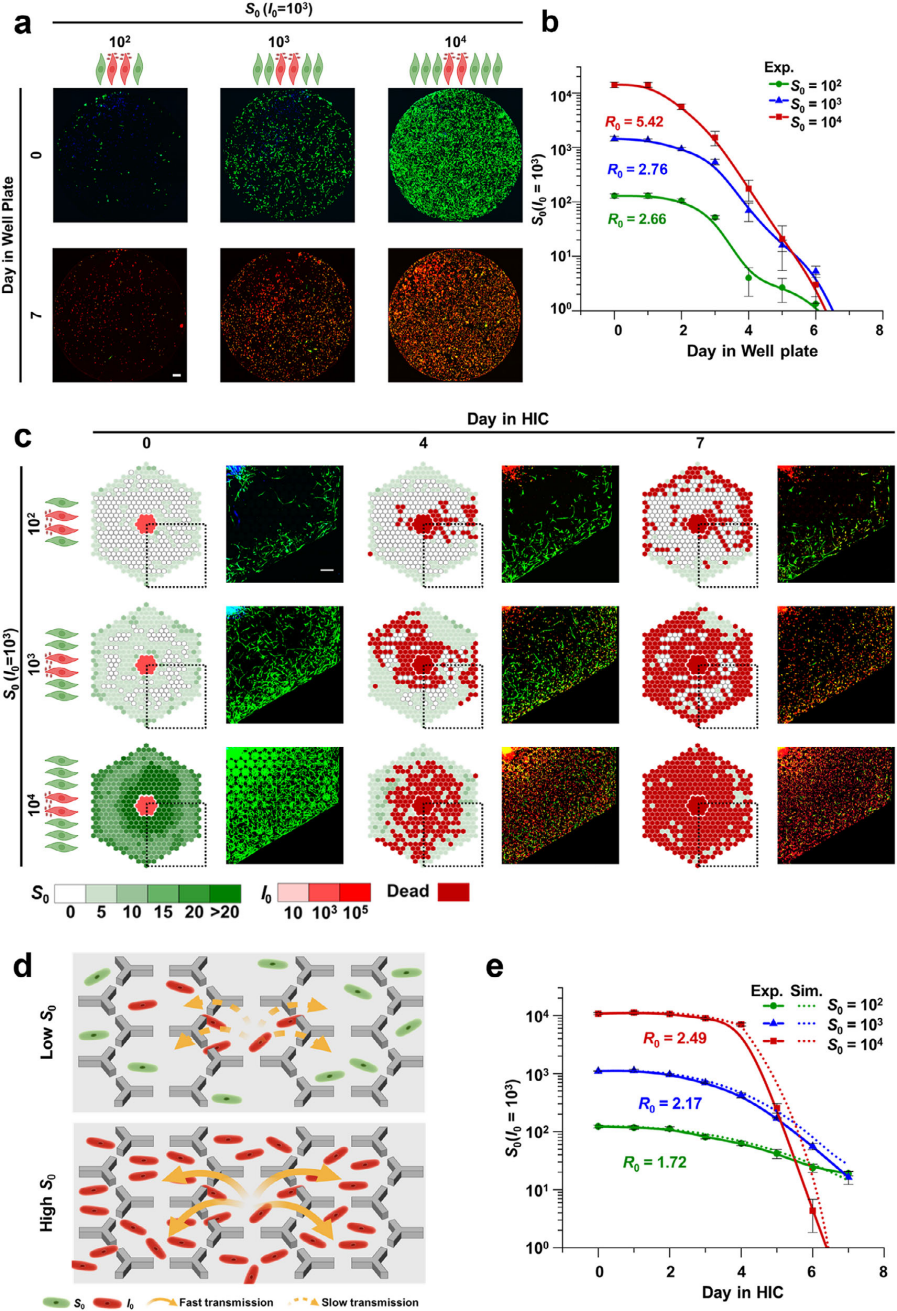
Effect of susceptible‐cell density (*S*_0_) on viral transmission in homogeneous wells and HIC. (a) 96‐well plates. Confocal images at day 0 and day 7 for *S*_0_ = 10^2^, 10^3^, 10^4^ at fixed *I*_0_ = 10^3^ (n = 3 wells per condition). Blue: initially infected cells (*I*_0_). Green: susceptible cells (*S*_0_). Red: dead cells. Cartoon icons indicate relative susceptible population size. (b) Quantification in wells. Time courses of remaining *S*_0_ over 7 days for each condition. *R*_0_ estimated by fitting *S*_0_‐depletion curves with a susceptible–infected–dead (SID) model [20]. (c) HIC spatial dynamics. Left: hexagonal‐lattice maps at days 0, 4, 7 showing chamberwise counts of *S*_0_ (green scale) and *I*_0_ (red scale); dead cells are indicated in dark red. Right: corresponding confocal images from the boxed lower‐right region. Blue: initially infected cells (*I*_0_). Green: susceptible cells (*S*_0_). Red: dead cells. (d) Schematic of contact‐mediated propagation in the HIC under low and high *S*_0_. Low *S*_0_ yields sparse encounters between initially infected (red) and susceptible (green) cells and slow front propagation, whereas high *S*_0_ increases contact opportunities and accelerates spread. (e) Quantification in HIC. *S*_0_ time courses for *S*_0_ = 10^2^, 10^3^, 10^4^ over 7 days. Solid lines, experiments; dotted lines, SEUID simulations. *R*_0_ values estimated from *S*_0_‐depletion fits. Scale bar, 500 µm. *Note*: The reference condition *S*_0_ = 10^3^ / *I*_0_ = 10^3^ in both the 96‐well plate and HIC experiments is shared with Figure 3 because it served as a common baseline condition in the same experimental run.

Urban centres during the early COVID‐19 outbreak highlighted how local susceptible‐host density shapes transmission trajectories [18]. To recapitulate density effects, we first established baseline behavior in homogeneous 96‐well plates by varying *S*_0_ = 10^2^, 10^3^, 10^4^ at fixed *I*_0_ = 10^3^ (Figure 2a), and in parallel applied the same conditions to HIC by seeding the epicentre and allowing spread across the compartmentalised network (Figure 2c). Seven‐day readouts quantified propagation in both formats.

In homogeneous wells, infection expanded rapidly and nearly all cells died by day 7 regardless of the initial *S*_0_ (Figure 2a). Time courses of remaining susceptible cells yielded *R*_0_ values of 2.66, 2.76, and 5.42 for *S*_0_ = 10^2^, 10^3^, 10^4^, respectively (Figure 2b), indicating that well‐mixed conditions amplify transmission and largely obscure any protective effect of low density. By contrast, HIC revealed strong *S*_0_ dependence: early spread was constrained by local contacts, higher *S*_0_ (10^3^–10^4^) accelerated propagation after a delay, and low *S*_0_ (10^2^) remained relatively protected. By day 4, devices at *S*_0_ = 10^4^ were predominantly infected, whereas *S*_0_ = 10^2^–10^3^ showed substantially fewer infected chambers; by day 7, widespread infection persisted at higher densities while many low‐density chambers remained uninfected (Figure 2c; Movies S1–S3). Robustness was observed with MRC‐5/HCoV‐OC43 and Huh‐7 (human hepatoma)/HCoV‐229E (Figure S1).

Comparison with SEUID simulations showed close agreement on HIC at *S*_0_ = 10^2^–10^4^, with *R*_0_ ≈ 1.72, 2.17, and 2.49 (Figure 2e). These data support a contact‐centred mechanism whereby increasing *S*_0_ increases the probability of infectious cell‐cell encounters (effective contact rate, *β*). Consistent with this interpretation, reducing cell motility by focal adhesion kinase (FAK) knockdown [19] without changing *S*_0_ delayed outward spread and attenuated transmission on HIC, while producing only modest mitigation in homogeneous wells (Figure S2, S3; Movies S4–S6). Together, the results indicate that spatial compartmentalisation exposes density‐ and contact‐limited dynamics that are masked in well‐mixed environments, with *S*_0_ and contact opportunities jointly determining effective transmission. We acknowledge, however, that cell proliferation during the 7‐day culture period may also contribute to local cell redistribution and cannot be completely distinguished from infection‐driven spread in this in vitro model.

### 2.2 Initial Infection Density (*I*_0_) Tunes Outbreak Onset in Structured Networks

We modelled scenarios from small clusters to large outbreaks by varying the initial infected population size (*I*_0_), reflecting early COVID‐19 superspreading linked to a large religious gathering in South Korea [10].

**FIGURE 3:**
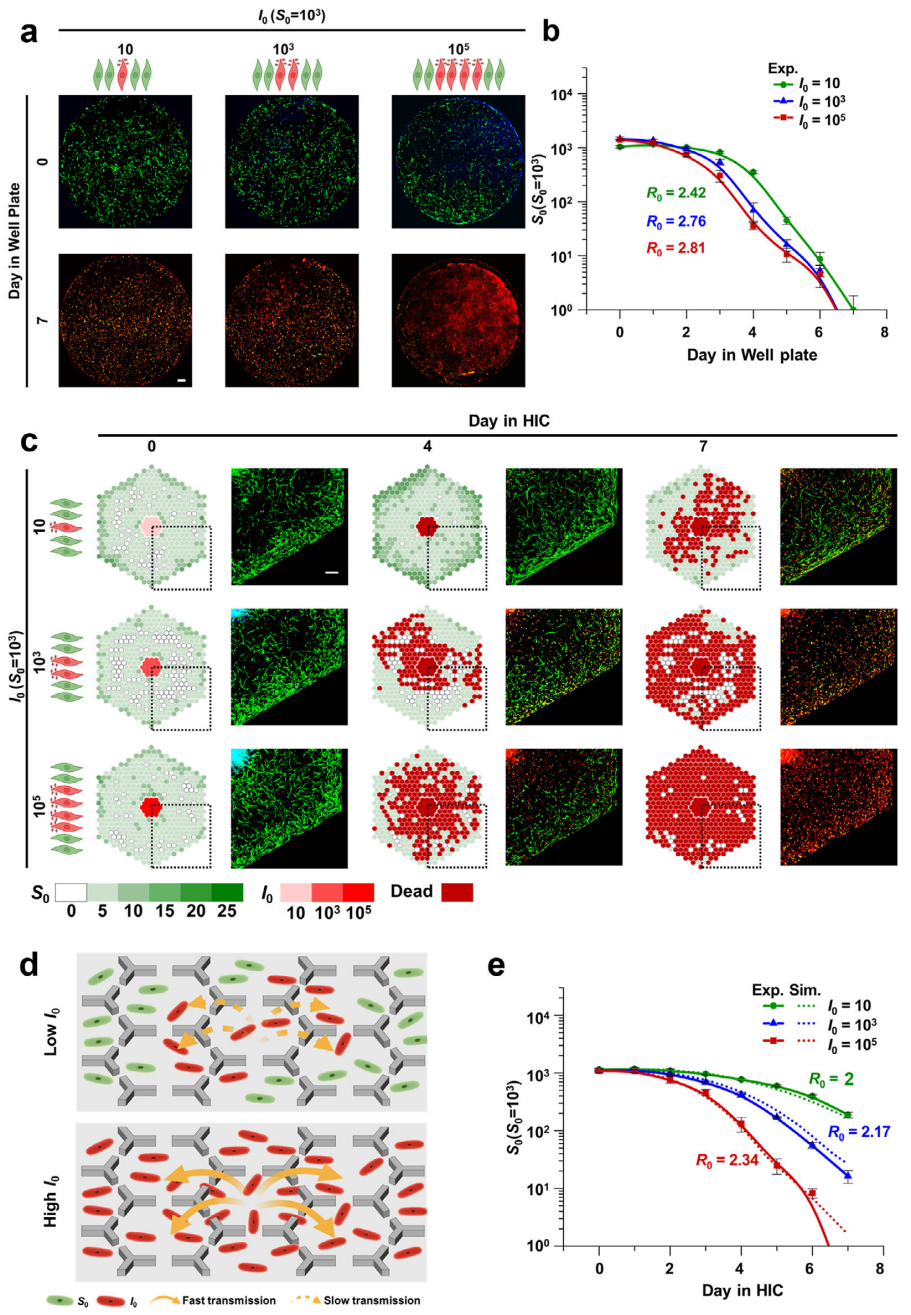
Initial infection density (*I*_0_) tunes outbreak onset in homogeneous wells and HIC. (a) 96‐well plates. Confocal images at day 0 and day 7 for fixed *S*_0_ = 10^3^ and varying *I*_0_ = 10, 10^3^, and 10^5^ (n = 3 wells per condition). Blue: initially infected cells (*I*_0_); green: susceptible cells (*S*_0_); red: dead cells. Cartoon icons indicate relative *I*_0_. (b) Quantification in wells. Time courses of remaining *S*_0_ over 7 days; *R*_0_ is estimated from the experimentally obtained *S*_0_‐depletion curves. (c) HIC spatial dynamics. Left: hexagonal‐lattice maps at days 0, 4, 7 showing chamberwise counts of *S*_0_ (green scale) and *I*_0_ (red scale); dead cells indicated in dark red. Right: matching confocal images from the boxed lower‐right region (n = 3 devices per condition). Blue: initially infected cells (*I*_0_); green: susceptible cells (*S*_0_); red: dead cells. (d) Contact‐based interpretation. Low *I*_0_ seeds few early foci and lengthens the lag; high *I*_0_ seeds many foci, shortening the lag and advancing onset. (e) Quantification in HIC. Time courses of remaining *S_0_* for *I*_0_ = 10, 10^3^, and 10^5^, with experimental data (solid lines) and SEUID simulations (dotted lines). *R_0_* values were derived from the experimentally obtained *S_0_*‐depletion curves. Scale bar, 500 µm. *Note*: The reference condition *S*_0_ = 10^3^ / *I*_0_ = 10^3^ in both the 96‐well plate and HIC experiments is shared with Figure 2 because it was obtained from the same experimental run and served as a common baseline condition.

We first examined how *I*_0_ influences spread in a homogeneous setting by inoculating 96‐well plates with *I*_0_ = 10, 10^3^, or 10^5^ at fixed *S*_0_ = 10^3^. Across all *I*_0_ values, wells exhibited rapid spread: substantial involvement by day 4 and near‐complete depletion of *S*_0_ by day 7 (Figure 3a). Quantifying *S*_0_ over time and fitting a SID model yielded *R*_0_ = 2.42, 2.76, and 2.81 for *I*_0_ = 10, 10^3^, and 10^5^, respectively (Figure 3b), indicating that the well‐mixed environment strongly amplifies transmission and is only weakly sensitive to initial infection density.

We next tested the same seeding conditions in the HIC with *I*_0_ = 10, 10^3^, 10^5^ (Figure 3c). Low *I*_0_ produced slow, localised spread with no detectable infection until day 7, whereas higher *I*_0_ increased early exposure frequency and accelerated dissemination, yielding extensive spread by day 4 and near‐complete loss of *S*_0_ by day 7. Once initiated, the *I*_0_ = 10 case propagated at a rate comparable to higher inocula, producing a similar final attack size (Movies S7, S8). The trend was robust across MRC‐5/HCoV‐OC43 and Huh‐7/HCoV‐229E experiments (Figure S4). Contact‐based reasoning (Figure 3d): low *I*_0_ starts few clusters and lengthens the delay before spread takes off; high *I*_0_ starts many clusters, shortening the delay and bringing earlier onset. SEUID simulations recapitulated the HIC data, with *R*_0_ declining from 2.34 to 2 as *I*_0_ decreased (Figure 3e). In contrast, well‐mixed controls produced higher *R*_0_ (2.42, 2.76, 2.81) with weak sensitivity to *I*_0_ (Figure 3b), masking the early‐phase delay revealed by the structured HIC network. Thus, while higher *I*_0_ accelerates early spread, it chiefly tunes outbreak onset (lag duration), with structured networks exposing early‐phase delays that are obscured in well‐mixed settings.

To further evaluate the physiological relevance and application potential of HIC, we performed additional *I*_0_‐focused validation experiments. First, we repeated the *I*_0_‐density transmission experiment using primary human lung fibroblasts. Similar to the results obtained with established cell lines, primary cells showed rapid transmission in homogeneous 96‐well plates, whereas HIC resolved *I*_0_‐dependent differences in outbreak onset and spatial propagation (Figure S13). This result indicates that the structured‐transmission behavior captured by HIC is not restricted to established cell lines, but can also be reproduced in primary human lung cells, supporting the physiological relevance of the platform.

In addition, to explore the potential use of HIC for early intervention testing, we pretreated I_0_ cells with imipramine, a reported inhibitor of coronavirus entry and intracellular trafficking, before loading them into the HIC epicentre, while the pre‐seeded susceptible population was maintained in normal medium. This targeted treatment delayed viral spread in HIC and reduced *R_0_* compared with untreated and dimethyl sulfoxide (DMSO) vehicle controls, whereas 96‐well controls showed rapid cell death from day 4 and retained high *R_0_* values regardless of treatment (Figure S14). These findings suggest that HIC can reveal intervention‐dependent delays in spatial transmission that are masked in well‐mixed culture.

### 2.3 Non‐Susceptible Fraction (*U*_0_) Establishes Herd‐Immunity Thresholds in Structured Networks

Herd immunity depends on the fraction of immune (or otherwise protected) individuals and, in practice, on vaccine coverage and effectiveness [21, 22]. In our framework, this protection is operationalised as a non‐susceptible fraction (*U*_0_) generated by APN knockdown in MRC‐5, rendering them refractory to infection by HCoV‐229E [16]. The remaining APN‐intact cells constitute *S*_0_.

**FIGURE 4:**
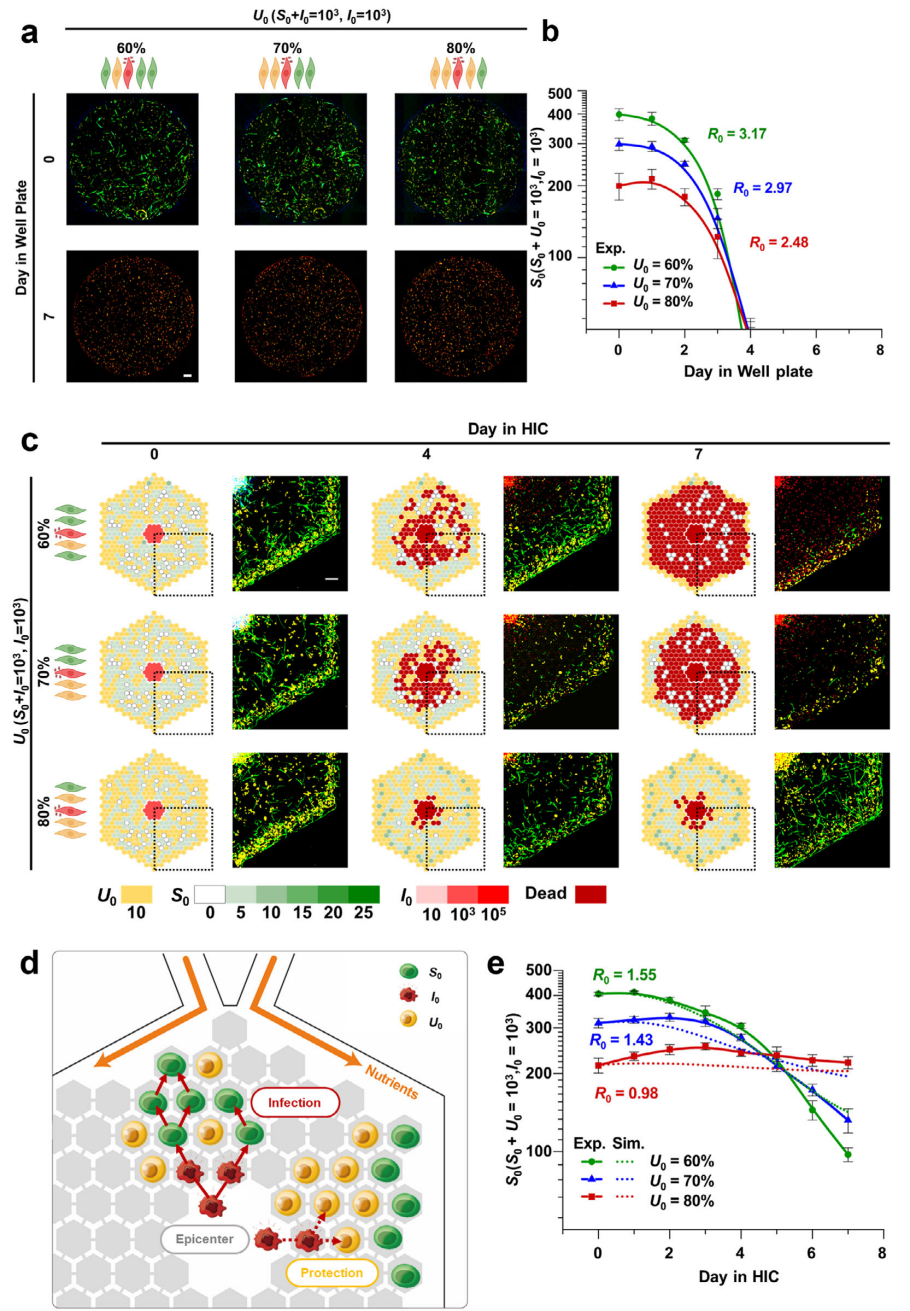
Effect of the non‐susceptible fraction (*U*_0_) on virus transmission in homogeneous wells and HIC. (a) 96‐well plates. Confocal images at days 0 and 7 for fixed *S*_0_ + *U*_0_ = 10^3^ and *I*_0_ = 10^3^, with *U*_0_ = 60%, 70%, or 80% of the 10^3^ uninfected cells (n = 3 wells per condition). Blue: initially infected (*I*_0_); green: susceptible (*S*_0_); yellow: non‐susceptible (*U*_0_); red: dead cells. Cartoon icons indicate the *U*_0_ proportion. (b) Quantification in wells. Time courses of remaining *S*_0_ over 7 days; *R*_0_ was estimated from the experimentally obtained *S*_0_‐depletion curves. (c) HIC spatial dynamics. Left: hexagonal‐lattice maps at days 0, 4, 7 showing chamberwise counts of *S*_0_ (green scale), *I*_0_ (red scale), and *U*_0_ (yellow). Right: corresponding confocal images from the boxed lower‐right region (n = 3 devices per condition). Blue: initially infected (*I*_0_); green: susceptible (*S*_0_); yellow: non‐susceptible (*U*_0_); red: dead cells. (d) Contact‐based interpretation. Low *U*_0_ leaves many permissive contact paths and permits extensive spread; high *U*_0_ sparsifies the contact graph, shielding susceptible clusters and breaking inter‐chamber transmission. (e) Quantification in HIC. Time courses of remaining *S*_0_ for *U*_0_ = 60%, 70%, and 80% with experimental (solid) and SEUID simulation (dotted) fits; *R*_0_ values were derived from *S*_0_‐depletion curves. Scale bar: 500 µm.

As a homogeneous‐mixing control, we first benchmarked transmission in conventional 96‐well plates, which eliminate spatial compartmentalization while maintaining the same virus–host interactions as the HIC, using *U*_0_ = 60%, 70%, 80% at fixed *S*_0_ + *U*_0_ = 10^3^ and *I*_0_ = 10^3^. Across all *U*_0_ levels, wells showed rapid spread: *S*_0_ was substantially infected by day 4 and fully depleted by day 7 (Figure 4a). The experimentally obtained *S*_0_ depletion curves yielded *R*_0_ = 3.17, 2.97, 2.48 for *U*_0_ = 60%, 70%, 80%, respectively (Figure 4b), indicating that well‐mixed environments fail to establish herd immunity even at high *U*_0_.

We then applied the same *U*_0_ fractions to HIC (Figure 4c). In HIC, *U*_0_ **=** 60–70% produced widespread infection by day 7, whereas *U*_0_ **=** 80% left more than half of *S*_0_ uninfected, with spread largely confined to the high‐contact epicentre and strongly attenuated peripherally (Figure 4c, Movies S9–S11). Similar trends were observed when CASD1 (CAS1 domain‐containing protein 1)‐knockdown MRC‐5 cells served as *U*_0_ (Figure S5a,b). CASD1 is a sialate O‐acetyltransferase that generates 9‐O‐acetylated sialic acid, the attachment moiety for HCoV‐OC43. Similar results were also obtained when APN‐knockdown Huh‐7 cells were used for HCoV‐229E (Figure S5c,d).

Mechanistically (Figure 4d), low *U*_0_ leaves many permissive contact paths, whereas high *U*_0_ sparsifies the contact graph, shielding susceptible clusters and breaking inter‐chamber relay. Quantitatively, increasing *U*_0_ from 60% → 80% reduced *R*_0_ from 1.55 → 0.98 in HIC, closely matching SEUID predictions; at 80% *U*_0_ infection of *S*_0_ was effectively halted by day 7 (Figure 4e). By contrast, well‐mixed plates retained *R*_0_ > 2.48 even at 80% *U*_0_ (Figure 4b), precluding herd immunity. Thus, herd‐immunity thresholds depend on both the protected fraction and contact topology: spatial compartmentalisation amplifies the protective effect of *U*_0_, enabling outbreak suppression at lower immune fractions than in well‐mixed settings. For modelling, treating *U*_0_ as a parameter that sparsifies the contact kernel provides a principled route to predicting thresholds in structured populations.

The herd‐immunity threshold identified in the HIC is specific to the present experimental configuration and should not be interpreted as a universal threshold for human populations. Rather, the platform is intended to experimentally demonstrate how spatial contact topology and the proportion of protected units jointly influence transmission dynamics under controlled conditions. Future studies incorporating more complex and heterogeneous contact networks may further improve its translational relevance.

## 3 Conclusion

We established a structure‐aware microfluidic platform—HIC—that quantitatively links population composition and contact mechanics to coronavirus transmission in a 444‐chamber hexagonal network. Increasing S_0_ or I_0_ elevated effective encounter rates and accelerated spread, whereas increasing U_0_ progressively attenuated transmission and, at 80% of the fixed uninfected pool, produced a herd‐immunity–like collapse that prevented network‐wide percolation. Reducing cell motility via FAK knockdown further confirmed that the frequency of physical cell–cell contacts is a direct driver of transmission, and that spatial isolation is a key regulator of intervention efficacy—dependencies that are largely masked in well‐mixed culture wells.

By fitting susceptible‐cell depletion trajectories to estimate *R*_0_ and comparing them with SEUID simulations, HIC couples directly observable infection fronts to mechanism‐aware modelling, improving the interpretability of density, seeding, immunity, and motility effects in structured populations. While the present chip uses a limited set of host cell types and a diffusion‐dominated 2D geometry, the framework is readily extensible to multi‐cell and immune co‐cultures, in situ sensing, and models with contact kernels that explicitly depend on density, motility, and spatial topology. Collectively, these results position HIC as a low‐barrier, scalable testbed for structure‐aware forecasting and for scenario testing of antivirals, vaccination coverage, and contact‐modulating strategies in preparation for future outbreaks.

## 4 Experimental Section

### 4.1 Cell Culture and Viruses

The MRC‐5 lung fibroblast and Huh‐7 human hepatoma cells were purchased from the Korean Cell Line Bank (Seoul, Korea). Green fluorescent protein (GFP)‐labelled MRC‐5 (GFP‐MRC‐5) and Huh‐7 (GFP‐Huh‐7) cells were purchased from Lugen Sci Co., Ltd. (Bucheon, Korea). The cells were maintained in minimum essential medium (MEM) (HyClone Laboratories, Logan, UT, USA) and Roswell Park Memorial Institute (RPMI) 1640 medium (HyClone Laboratories) supplemented with 10% (v/v) foetal bovine serum (FBS) (HyClone Laboratories), 100 units penicillin mL^−1^ (Life Technologies, Carlsbad, CA, USA), 100 µg streptomycin mL^−1^ (Life Technologies), 25 mM sodium bicarbonate (Sigma‐Aldrich), and 25 mM hydroxyethyl piperazine ethane sulfonic acid (HEPES) (Life Technologies) at 37°C with 5% CO_2_. The HCoV strains (229E and OC43) were provided by the Korea Research Institute of Bioscience and Biotechnology (KRIBB) (Daejeon, Korea).

### 4.2 Virus Propagation and Measurement of the 50% Tissue Culture Infective Dose (TCID_50_)

Virus propagation and measurement of TCID_50_ values were performed as described previously [23].

### 4.3 Cytopathic Effect (CPE)

MRC‐5 cells (10^4^ cells per well) were seeded in 96‐well plates and incubated overnight at 37°C with 5% CO_2_. The cells were washed with phosphate‐buffered saline (PBS) and inoculated with HCoV‐229E viruses at 10^−3^ to 10^3^ TCID_50_ mL^−1^ diluted in modified MEM with 2% FBS at 33°C in a 5% CO_2_ incubator [24, 25]. After 3 days of incubation, CPE, such as rounding and aggregation of cells, was monitored (Figure S6).

### 4.4 Preparation of Infected Cells (*I_0_*)

To generate infected cells, host (MRC‐5) cells (5 × 10^5^ cells) were seeded in a T‐75 flask (SPL Life Sciences, Pocheon, Korea) and incubated overnight at 37°C with 5% CO_2_. The cells were washed with PBS and inoculated with HCoV‐229E at 100 TCID_50_ mL^−1^ in MEM at 33°C with 5% CO_2_. On the next day, infected cells were labelled with Hoechst 33342 (2’‐[4‐ethoxyphenyl]‐5‐[4‐methyl‐1‐piperazinyl]‐2.5‐bi‐1H‐benzimidazole trihydrochloride trihydrate) (Sigma‐Aldrich), which stains the nuclei of living cells, before seeding into the epicentre (Figure S7).

### 4.5 Preparation of *U_0_* and Low‐Motility *S_0_* cells

GFP‐MRC‐5 cells were seeded in 6‐well plates at 2 × 10^5^ cells/well and cultured overnight in MEM containing 10% FBS. To knock down APN, CASD1, and FAK, the respective cells were transfected with 100 nM AccuTarget siRNA (Table 1) using Lipofectamine RNAiMAX transfection reagent (Invitrogen) according to the manufacturer's protocol. As a negative control, parallel wells received 100 nM AccuTarget negative control siRNA (Bioneer Co., Daejeon, Korea). After 48 h of transfection, KD efficiency was verified using quantitative reverse transcription‐PCR (qRT‐PCR) and western blot before seeding these APN or CASD1‐KD cells as *U*_0_ and FAK‐KD cells as low‐motility *S*_0_ in the HIC experiments.

**TABLE 1:** siRNA sequences.

	Sense	Antisense
**FAK siRNA**	5’‐ AAC CAC CUG GGC CAG UAU UAU ‐3’	5’‐ AUA AUA CUG GCC CAG GUG GUU ‐3’
**APN siRNA**	5’‐GCA GCA GAU CUG UAU AUU U‐3’	5’‐AAA UAU ACA GAU CUG C‐3’
**CASD1 siRNA**	5’‐GGA UAU GCC CGU UCA GUU U‐3’	5’‐ AAA CUG AAC GGG CAU AUC C ‐3’

### 4.6 Evaluation of the Transfection Efficiency

Total RNA was extracted and purified from the cells using a spin column kit (Zymo Research, Irvine, CA, USA). Complementary DNA (cDNA) fragments were synthesised from the extracted RNA using a first‐strand cDNA synthesis system (LeGene Biosciences, San Diego, CA, USA). The primer sequences used were designed by Bioneer Co. (Table 2). The increase in amplified DNA was measured using iQ SYBR Green premix (Bio‐Rad, Hercules, CA, USA).

**TABLE 2:** Primer sequences.

	Sense	Antisense
**FAK**	5’‐GGC TGT CAT CGA GAT GTC CA ‐3’	5’‐TCT CAT CCA CCG TGG CTA GT‐3’
**APN**	5’‐GTG ATG GCA GTG GAT GCA CAG CTT‐3’	5’‐CCT GTC CGA GGA CTG TA‐3’
**CASD1**	5’‐GTG GAT TTT CTG TGG CAT CC‐3’	5’‐AAG CGC TTC ACT GCT ACC AT‐3’
**GAPDH**	5’‐ AGG TGG TGA AGC AGG CGT CGG AGG G‐3’	5’‐CAA AGT GGT CGT TGA GGG‐3’

Western blotting was performed to confirm transfection efficiency. Cells were lysed using radioimmunoprecipitation assay (RIPA) buffer (Bio‐solution, Suwon, Korea), and proteins were extracted. The protein concentration in RIPA buffer was measured using Bradford reagent (Sigma). Proteins (30 µg) were separated using 10% SDS‐PAGE and then probed with antibodies (Figure S8).

For the first antibody treatment, anti‐CD13 (APN) antibody (ab108382; Abcam, Cambridge, UK) at a 1:5,000 dilution, CASD1 antibody (PA5‐69624) from Invitrogen (Waltham, MA, USA) at a 1:1,000 dilution, FAK antibody (AHO1272) from Invitrogen at a 1:500 dilution, and GAPDH antibody (Santa Cruz Biotechnology) at a 1:8,000 dilution were used. For the secondary antibody treatment, anti‐rabbit‐HRP antibody (Thermo Fisher) at a 1:10,000 dilution and anti‐mouse HRP antibody (ab205719; Abcam, Cambridge, UK) at a 1:5,000 dilution were used.

### 4.7 Evaluation of Virus Infectivity

MRC‐5 and APN‐KD MRC‐5 cells were seeded in 96‐well plates (Corning Inc., Corning, NY, USA) (10^4^ cells/well) and incubated overnight at 37°C with 5% CO_2_. The cells were washed with PBS and inoculated with HCoV‐229E at 10 to 10^3^ TCID_50_ mL^−1^ diluted in MEM with 2% FBS at 33°C with 5% CO_2_ for 3 days (Figure S9).

### 4.8 Measurement of Cell Migration Ability

MRC‐5 (Con) and FAK‐KD MRC‐5 cells (10^4^ cells/well) were seeded on a 6‐well plate (Corning Inc.). To observe the migration distance of cells, images of displacement with cells were obtained at 1 and 24 h in a live cell chamber (Live Cell Instrument, Seoul, Korea) at 37°C with 5% CO_2_ using a DeltaVision microscope (GE Healthcare) equipped with a CoolSnap HQ2 camera (Photometrics, Tucson, AZ, USA). Cell movement tracks were labelled, and total displacement (Figure S10; Movies S12–S15) was measured using ImageJ (NIH, Bethesda, MD, USA).

### 4.9 Fabrication of HIC

A previously developed chip [15, 26] was used to prepare the HIC (Figure S11). Briefly, a mould of HIC was first fabricated using a negative photoresist SU–8 (MicroChem Co., Westborough, MA, USA) spin‐coated on a 4‐inch wafer for 30 s (SU–8 2025, 2000 rpm, 40 µm thickness). The HIC was cast into polydimethylsiloxane (PDMS) (Sylgard 184, Dow Corning Co.) by soft lithography [27]. The HIC comprised a 444‐chamber microfluidic network (200 µm long and 40 µm deep) created using microchannels that were 30 µm wide. 5 µm‐high sidewalls were present at the edges of the microchambers to allow nutrient flow into the interior of the microchambers. Nutrients were perfused from the four reservoirs through two microchannels (150 µm wide) surrounding the chambers. A 700 µm‐diameter seeding port (‘epicentre’) was punched. The process of sterilising and coating the chips has been described previously [26].

### 4.10 Cell Loading Strategy for Establishing the Initial Spatial Distribution in HIC

To establish the initial spatial organization of the HIC, *S*_0_/*U*_0_ and *I*_0_ cells were loaded using different procedures. For *S*_0_ seeding, or *S*_0_/*U*_0_ seeding in the herd‐immunity experiments, gentle manual flow was applied through the epicentre inlet to distribute the cell suspension throughout the interconnected microchamber network. After loading, the HIC was incubated overnight to allow cell attachment and stabilization across the network. In contrast, *I*_0_ cells were intended to serve as a localized initial infection source. Therefore, *I*_0_ cells were loaded without applying manual flow; the infected‐cell suspension was gently placed in the epicentre and allowed to settle by gravity. This procedure maintained *I*_0_ cells in the central epicentre region at day 0, while *S*_0_ and *U*_0_ cells were distributed throughout the surrounding microchamber network. The initial distribution of each fluorescently labeled cell population was confirmed from day 0 stitched fluorescence images before longitudinal transmission analysis.

### 4.11 Effect of Susceptible Cell (*S_0_*) Density on Virus Transmission in HIC and a 96‐Well Plate

Before observing the virus transmission in the HIC, totals of 10^3^, 10^4^, and 10^5^ GFP‐MRC‐5 cells were suspended in 10 µL culture medium and 1 µL of cell suspension was injected via the epicentre using a 200 µL pipette tip (T‐200, Axygen; Union City, CA, USA), so that the final number of initial *S*_0_ was 10^2^, 10^3^, 10^4^ cells/HIC, respectively. Considering that the area of the well of a 96‐well plate is 1.6 times that of a chamber of the HIC, the final number of initial *S*_0_ in the 96‐well plate was set as 1.6 × 10^2^, 1.6 ×10^3^, 1.6 ×10^4^ cells/well to normalize the same cell density as that in the HIC. The HIC and a 96‐well plate were incubated overnight at 37°C with 5% CO_2_.

On the next day, *I*_0_ at 10^4^ cells/10 µL in a 10‐µL pipette tip (T‐300) were injected and flowed into the epicentre by gravity for virus transmission. The final number of *I*_0_ cells was 10^3^ cells/HIC and 1.6 × 10^3^ cells/well. The HIC and 96‐well plate containing both *S*_0_ and *I*_0_ cells were incubated at 33°C with 5% CO_2_ for 7 days. Each day, the medium was replaced with 200 µL of fresh culture medium. To generate the cell number data, optical and fluorescence images were obtained daily using a K1‐Fluo confocal microscope (Nanoscope Systems, Inc., Daejeon, Korea). We employed the K1‐array viewer to acquire stitched images across various channels at a 4× magnification. Images were processed using ImageJ. The number of live susceptible cells was manually counted from days 0 to 7.

### 4.12 Effect of *I_0_* Density on Virus Transmission in the HIC and a 96‐Well Plate

GFP‐MRC‐5 cells at 10^3^ cells/HIC and 1.6 × 10^3^ cells/well were seeded in the HIC and 96‐well plate and incubated overnight at 37°C with 5% CO_2_. The next day, *I*_0_ at 10^1^, 10^3^, and 10^5^ cells/HIC and 1.6 × 10^1^, 1.6 × 10^3^, 1.6 × 10^5^ cells/well were flowed into the epicentre by gravity. The HIC containing both *S*_0_ and *I*_0_ cells was incubated at 33°C with 5% CO_2_ for 7 days. The subsequent steps were the same as those described in the ‘Effect of *S*_0_ density on virus transmission in HIC’. For primary‐cell validation, the same *I*_0_‐density transmission experiment was repeated using primary human lung fibroblasts purchased from Lonza (Walkersville, MD, USA) and maintained in fibroblast basal medium (FBM) from Lonza, with the same seeding, infection, imaging, and 7‐day culture procedures described above.

### 4.13 Effect of *U_0_* Density on Virus Transmission in the HIC and a 96‐Well Plate

For herd immunity experiments, *S*_0_ (GFP‐MRC‐5) and *U*_0_ (APN‐KD MRC‐5) were mixed at a ratio of 4:6 to 2:8 with a total of 10^3^ cells/HIC and 1.6 × 10^3^ cells/well seeded into the HIC and 96‐well plate, respectively. To visualise *U*_0_, cells were labelled with the CellMask Deep Red Plasma Membrane Stain (Invitrogen, Waltham, MA, USA) according to the manufacturer's instructions before seeding. After 1 day of incubation, *I*_0_ at 10^4^ cells/10 µL in a 10‐µL pipet tip were injected and flowed into the epicentre by gravity for virus transmission, and 1.6 × 10^3^ cells/well were added as the *I*_0_ in the 96‐well plate.

### 4.14 Effect of Imipramine‐Treated *I_0_* Cells on Virus Transmission in the HIC and a 96‐Well Plate

To evaluate the applicability of HIC for early intervention testing, imipramine treatment was applied only to *I*_0_ before loading. *S*_0_ cells were seeded in the HIC and 96‐well plates using the same procedure described above and maintained overnight in normal culture medium without drug treatment. On the next day, HCoV‐229E‐infected *I*_0_ cells were prepared as described above and resuspended in culture medium containing imipramine. For vehicle controls, *I*_0_ cells were resuspended in culture medium containing 0.1% (v/v) of DMSO. Untreated control *I*_0_ cells were resuspended in normal culture medium. The *I*_0_ suspensions were then loaded into the HIC epicentre or added to 96‐well plates using the same loading procedure as described for the *I*_0_‐density experiment. The pre‐seeded *S*_0_ cells in the HIC were not directly exposed to imipramine or DMSO before *I*_0_ loading. After *I*_0_ loading, the HICs and 96‐well plates were incubated at 33°C with 5% CO_2_ for 7 days. Daily imaging, medium replacement, live/dead staining, *S*_0_ counting, and *R*_0_ estimation were performed as described above.

### 4.15 SEUID‐Based Computational Model of Virus Transmission

To describe virus transmission on the HIC platform, we developed a spatial model incorporating five cell states: susceptible (*S*), exposed (*E*; infected, but not yet infectious), non‐susceptible (*U*), infected (*I*), and dead (*D*). This model accounts for spatial confinement by indexing each microchamber as *I*, with virus transmission driven by local interactions and constrained interchamber migration. *S* and *U* cells undergo division at rate *γ*, while *I* cells do not divide and are assumed to die (death rate *µ*), precluding recovery.

The system is governed by the following equations:

(1) $$\frac{dS_{i}}{dt}=-\beta_{w}S_{i}I_{i}-\beta_{n}\sum_{j}^{n.n.}\sqrt{S_{i}}\sqrt{I_{j}}+\gamma S_{i}$$

(2) $$\frac{dE_{i}}{dt}=\beta_{w}S_{i}I_{i}+\beta_{n}\sum_{j}^{n.n.}\sqrt{S_{i}}\sqrt{I_{j}}-\alpha E_{i}$$

(3) $$\frac{dI_{i}}{dt}=\alpha E_{i}-\mu I_{i}$$

(4) $$\frac{dU_{i}}{dt}=\gamma U_{i}$$

(5) $$\frac{dD_{i}}{dt}=\mu I_{i}$$

here, *β_w_* and *β_n_* represent the intra‐chamber and nearest‐neighbour inter‐chamber contact rates, respectively. To reflect the suppressive effect of immune cells on contact probability in confined domains, these rates are modulated as: β_*w*_  =  β_0_(1 − *c*(*U_i_* − *U_cr_*)^*m*^) and β_*n*_  =  β_1_(1 − *c*(*U_i_* − *U_cr_*)^*m*^). The model was fitted to the experimental data using nonlinear least squares regression.

### 4.16 Calculation of *R_0_*

In the standard SID framework [17, 20], the basic reproduction number *R*_0_ is defined as $R_{0}=\frac{\beta }{\mu }\;,$where β is the transmission rate and μ is the death rate. In the present analysis, rather than deriving *R*_0_ from the microscopic parameters β and μ of a SID or SEUID model, we estimated it as an *effective* reproduction number from operational quantities obtained directly from the experimental data: $R_{0}^{eff}=\frac{\beta_{eff}}{\mu_{eff}}=\beta_{eff}\;\tau_{infec}$, where β_*eff*_ is the effective transmission rate and $\tau_{infec}=\frac{1}{\mu_{eff}}\;$ is the infectious period.

The effective transmission rate β_*eff*_ was operationalised as the average daily fractional decrease of *S*(*t*) over the first two days of spread: $\beta_{eff}=-\frac{1}{S_{0}}\frac{S\left(2^{nd}\;day\right)-S_{0}}{2\;days}$. The infectious period τ_*infec*_ was approximated as the time required for a detectable cytopathic effect (CPE) to appear at the microchamber level, including cell rounding, aggregation, loss of GFP‐positive cells, and the appearance of dead‐cell staining. We used τ_*infec* _ =  2 *days*, based on the time required for HCoV‐229E infection to produce an observable CPE.

### 4.17 Statistical Analysis

Statistical significance was determined using Student's *t*‐test. The results are presented as the mean ± standard deviation (S.D.). All quoted *p*‐values were two‐tailed, and differences were considered statistically significant at *p < 0.05, **p < 0.01, and ***p < 0.001.

## Author Contributions

**Zhenzhong Chen**: data curation, methodology, validation. **Narina Jung**: data curation, writing – original draft, software, methodology, investigation, formal analysis, writing – review and editing. **Sungsu Park**: writing – original draft, supervision, investigation, conceptualization, methodology, funding acquisition, validation, writing – review and editing, project administration, data curation. **Wanyoung Lim**: conceptualization, methodology, data curation, visualization, investigation. **Min‐Hyeok Kim**: data curation, validation, visualization, methodology, writing – review and editing, investigation. **Byung Mook Weon**: conceptualization, supervision, investigation. **Jiande Zhang**: methodology, data curation, writing – original draft, investigation, formal analysis, visualization.

## Funding

This work was supported by National Research Foundation of Korea (NRF) grants funded by the Ministry of Science and ICT (RS‐2023‐00234581) and the Ministry of Education (RS‐2019‐NR040077), and by KIAS individual grant (PG085701) at Korea Institute for Advanced Study.

## Conflicts of Interest

The authors declare no conflicts of interest.

## Supporting information




**Supporting File 1**: advs77383‐sup‐0001‐SuppMat.docx.

**Supporting File 2**: advs77383‐sup‐0002‐SuppMat.zip.

## Data Availability

The data that support the findings of this study are available in the supplementary material of this article.
