# Supplementary material for: Decoding Viral Transmission Dynamics in Structured Populations Using a Microfluidic Herd‐Immunity‐on‐a‐Chip Platform
Source: Adv Sci (Weinh). 2026 Sep 3:e77383. Online ahead of print. doi: 10.1002/advs.77383 (PMC13542517; doi:10.1002/advs.77383)
Supplement: Supplementary file 1 [file ADVS-9999-e77383-s002.docx]

Supporting Information

**Decoding viral transmission dynamics in structured populations using a microfluidic Herd-Immunity-on-a-Chip platform**

*Jiande Zhang^+^, Narina Jung^+^, Min-Hyeok Kim****^+^,*** *Wanyoung Lim****^+^****, Zhenzhong Chen, Byung Mook Weon*, and Sungsu Park**

**This PDF file includes:**

Fig. S1-S14

Movies S1-S15

**Supplementary Figure S1. Susceptible cell density controls coronavirus transmission in Herd-Immunity-on-a-Chip (HIC) across two virus–host models (fixed *I*_0_ = 10³).** (A) HCoV-OC43–MRC-5 model. Susceptible MRC-5 cells were seeded on HIC at three densities (*S*_0_ = 10², 10³, 10⁴ cells per device). A fixed number of initially infected cells (*I*_0_ = 10³; OC43-infected MRC-5) was then introduced into the central epicentre by gravity flow. Devices were incubated for 7 days at 33 °C and 5% CO₂, with daily medium exchange. Whole-device images were acquired daily by a K1-Fluo confocal microscope (Nanoscope Systems, Inc., Daejeon, Korea) at 4× with tile-stitching; fluorescence channels denote susceptible (green), infected (blue), and dead (red) cells. MRC-5 cells present 9-O-acetylated sialic acids, the attachment determinant required for OC43 entry.^[1, 2]^ (in triplicate for HICs per condition; scale bar = 500 μm). (B) Time courses of remaining *S*_0_ under the same *I*_0_ conditions in the HIC during 7-day OC43 infection. *R*_0_ values estimated from the susceptible cell depletion curves revealed a strong dependence on *S*_0_. (C) 229E–Huh-7 model. Susceptible Huh-7 cells (*S*_0_ = 10², 10³, 10⁴ per device) were patterned on HIC and exposed to a fixed *I*_0_ = 10³ (229E-infected cells seeded at the epicentre). Culture and imaging followed the same 7-day schedule as in (A). Huh-7 cells express aminopeptidase N (APN), the canonical entry receptor for HCoV-229E.^[3]^ (in triplicate for 3 HICs per condition; scale bar = 500 μm). (D) Time courses of remaining *S*_0_ under the same *I*_0_ conditions in the well plate during 7-day 229E infection. *R*_0_ values derived from SID model fitting of susceptible cell depletion revealed a strong dependence on *S*_0_.

**Supplementary Figure S2. HCoV-229E transmission in HIC versus 96-well plates with different densities of low-motility susceptible cells (focal adhesion kinase: FAK)-knockdown (KD) *S*_0_; fixed *I*_0_ = 10³).** (A) HIC. Low-motility FAK-KD MRC-5 susceptible cells (*S*_0_ = 10², 10³, 10⁴ cells per device) were patterned across the chip’s 444 interconnected hexagonal microchambers. FAK AccuTarget™ siRNA (Bioneer Co., Daejeon, Korea) was transfected using Lipofectamine RNAiMAX (Invitrogen, Carlsbad, CA) according to the manufacturer’s protocol 48 h prior to HIC loading. The siRNA sequences for FAK is shown in **Table 1**, and the primer sequences are shown in **Table 2**. A fixed inoculum of *I*_0_ = 10³ was gravity-loaded into the central epicentre. Chips were maintained for 7 days at 33 °C and 5% CO₂, with daily medium exchange. Whole-device images were acquired daily by a K1-Fluo confocal microscope at 4× with tile-stitching; fluorescence channels denote susceptible (green), infected (blue), and dead (red) cells; spatial infection maps mark regions of interest. For each hexagonal chamber, the number of *S*_0_ cells is highlighted in green (six intensity levels), and that of *I*_0_ is indicated in red (three intensity levels). (in triplicate for HICs per condition; scale bar = 500 μm). (B) 96-well plate. The same FAK-KD *S*_0_ densities (10², 10³, 10⁴ per well; scaled to match HIC area) were combined with *I*_0_ = 10³ and cultured under the same conditions. Fluorescence images were collected daily. (in triplicate for wells per condition; scale bar = 500 μm). (C) HIC. Time courses of remaining low-motility *S*_0_ under the same *I*_0_ conditions in the HIC during 7-day 229E infection. *R*_0_ values estimated from the susceptible cell depletion curves revealed a strong dependence on low-motility *S*_0_. (D) 96-well plate. Time courses of remaining low-motility *S*_0_ under the same *I*_0_ conditions in the well plate during 7-day 229E infection. *R*_0_ values derived from the susceptible cell depletion curves revealed a strong dependence on low-motility *S*_0_.

**Supplementary Figure S3.** **HCoV-OC43 transmission in HIC with different densities of low-motility susceptible cells (FAK-KD *S*_0_; fixed *I*_0_ = 10³).** (A) The effect of motility on virus transmission was assessed by transfecting MRC-5 susceptible cells with siRNA targeting FAK to reduce cell motility. A total of 10³ FAK-KD *S*_0_ were seeded per HIC, and 10³ *I*_0_ were subsequently seeded into the central epicentre by gravity on day 1. The chips were maintained at 33 °C with 5% CO₂ for 7 days. Whole-device images were acquired daily by a K1-Fluo confocal microscope at 4× with tile-stitching; fluorescence channels denote susceptible (green), infected (blue), and dead (red) cells. (in triplicate for HICs per condition; scale bar = 500 μm). (B) Time courses of remaining low-motility *S*_0_ under the same *I*_0_ conditions in the HIC during 7-day OC43 infection. *R*_0_ values estimated from the susceptible cell depletion curves revealed a strong dependence on low-motility *S*_0_.

**Supplementary Figure S4.** **Initially infected cell density controls coronavirus transmission in HIC across two virus–host models (fixed *S*_0_ = 10³).** (A) OC43–MRC-5 model. Susceptible MRC-5 cells were seeded on HIC at *S*_0_ = 10³ cells per device. A fixed-area inoculum of initially infected cells (*I*_0_ = 10, 10³, 10⁵) was gravity-loaded into the central epicentre on the following day. Devices were maintained for 7 days at 33 °C and 5% CO₂, with daily medium exchange. Whole-device images were acquired daily by a K1-Fluo confocal microscope at 4× with tile-stitching; fluorescence channels denote susceptible (green), infected (blue), and dead (red) cells. (in triplicate for HICs per condition; scale bar = 500 μm). (B) Time courses of remaining *S*_0_ under the same *I*_0_ conditions in the HIC during 7-day OC43 infection. *R*_0_ values estimated from the susceptible cell depletion curves revealed a strong dependence on *I*_0_. (C) 229E–Huh-7 model. Susceptible Huh-7 cells were seeded at *S*_0_ = 10³ per device. *I*_0_ = 10, 10³, 10⁵ were introduced centrally as in (A). Culture and imaging followed the same 7-day schedule. (in triplicate for HICs per condition; scale bar = 500 μm). (D) Time courses of remaining *S*_0_ under the same *I*_0_ conditions in the HIC during 7-day 229E infection. *R*_0_ values derived from SID model fitting of susceptible cell depletion revealed a strong dependence on *I*_0_.

**Supplementary Figure S5. Non-susceptible fraction on transmission in HIC (*U*_0_ = 60–80% at fixed *S*_0_+*U*_0_ = 10³ and *I*_0_ = 10³).** (A)OC43–MRC-5 model. Total uninfected cells per device were fixed at 10³ (*S*_0_ + *U*_0_ = 10³) and mixed to yield *U*_0_ = 60%, 70%, or 80%. *U*_0_ were generated by siRNA knockdown of CASD1 in MRC-5 cells. CASD1 siRNA was transfected using Lipofectamine RNAiMAX according to the manufacturer’s protocol 48 h prior to HIC loading. The sequence of CASD1 is shown in **Tab. 1**, and the sequence of primer is shown in **Tab. 2**. On day 1, *I*_0_ = 10³ OC43-infected cells were gravity-loaded into the central epicentre. Devices were cultured for 7 days at 33 °C and 5% CO₂, with daily medium exchange. Whole-device fluorescence images were acquired daily by a K1-Fluo confocal microscope at 4× with tile-stitching; fluorescence channels denote susceptible (green), yellow (non-susceptible), infected (blue), and dead (red) cells. (in triplicate for HICs per condition; scale bar = 500 μm). (B) Time courses of remaining *S*_0_ under the same *I*_0_ conditions in the HIC during 7-day OC43 infection. *R*_0_ values estimated from the susceptible cell depletion curves revealed a strong dependence on *U*_0_. (C) 229E–Huh-7 model. Using the same design, Huh-7 cells were seeded with *U*_0_ = 60%, 70%, 80% at constant *S*_0_ + *U*_0_ = 10³ per device, and *I*_0_ = 10³ HCoV-229E-infected cells were introduced centrally. *U*_0_ were produced by siRNA knockdown of aminopeptidase N (APN), the entry receptor for 229E. The sequence of APN is shown in **Tab. 1**, and the sequence of primer is shown in **Tab. 2**. Culture and imaging followed the schedule in (A). (in triplicate for HICs per condition; scale bar = 500 μm). (D) Time courses of remaining *S*_0_ under the same *U*_0_ conditions in the HIC during 7-day 229E infection. *R*_0_ values estimated from the susceptible cell depletion curves revealed a strong dependence on *U*_0_.

**Supplementary Figure S6. Morphological changes of MRC-5 cells after human coronavirus infection. (A)** MRC-5 cells were inoculated with 100 μL of HCoV-229E at different concentrations (10^-3^ to 10^3^ TCID_50_/mL) and incubated at 37 °C with 5% CO_2_ for 3 days. **(B)** MRC-5 cells were inoculated with 100 μL of HCoV-OC43 at different concentrations (10^-3^ to 10^3^ TCID_50_/mL) and incubated at 37 °C with 5% CO_2_ for 3 days. Images were taken with a K1-Fluo confocal microscope with 4× magnification. Scale bar = 100 μm.

**Supplementary Figure S7. Fluorescent images of the epicentre in HIC after seeding of initially infected cells.** Various numbers of infected cells (10^1^, 10^3^, and 10^5^) were stained with Hoechst 33342 (blue) for 5 min and seeded into the epicentre. Images were taken with a K1-Fluo confocal microscope with 4× magnification. Scale bar = 200 μm.

**Supplementary Figure S8. Validation of knockdown (KD) efficiency for APN, CASD1, and FAK.** (A, B, C) Quantitative reverse transcription-PCR (qRT-PCR) analysis of mRNA expression levels of aminopeptidase N (APN), CAS 1 domain containing 1 (CASD1), and focal adhesion kinase (FAK) in control (NC) and siRNA-mediated KD groups. GAPDH was used as an internal control (in triplicate for per group). Data are presented as mean ± SD. (D, E, F) Western blot analysis confirming reduced protein expression of APN, CASD1, and FAK in KD cells compared to NC. GAPDH was used as a loading control. Statistical significance was determined by Student’s *t*-test, ****p* < 0.001.

**Supplementary Figure S9. Confirming virus infectivity. (A)** Morphological changes of MRC-5 (CON) and APN-KD MRC-5 cells after HCoV-229E infection. MRC-5 cells were inoculated with 100 μL of HCoV-229E at different concentrations (10^1^ to 10^3^ TCID_50_/mL) and incubated at 37 °C with 5% CO_2_ for 3 days. **(B)** Morphological changes of CON and siCASD1 (CASD1-KD MRC-5) cells after HCoV-OC43 infection. MRC-5 cells were inoculated with 100 μL of HCoV-OC43 at different concentrations (10^1^ to 10^3^ TCID_50_/mL) and incubated at 37 °C with 5% CO_2_ for 3 days. Images taken by a K1-Fluo confocal microscope with 4× magnification. Scale bar = 100 μm.

**Supplementary Figure S10. Confirming the cell migration ability. (A)** Migration ability changes of MRC-5 (Control; CON) and FAK-KD MRC-5 cells after attaching on a 6-well plate at 37 °C and 5% CO_2_ for 24 hours. Image taken by DeltaVision microscope (GE Healthcare) with 4× magnification. **(B)** Related migration of WT and FAK-KD MRC-5 cells (in decuplicate for per group). Statistical significance was determined by Student’s *t*-test, ****p* < 0.001. Scale bar = 50 μm.

**Supplementary Figure S11. Geometry of the HIC and key dimensions. (A)** Plan view with unit-cell inset. The HIC comprises 444 interconnected hexagonal microchambers (200 µm across flats; depth 40 µm) connected by 30 µm-wide microchannels. Peripheral sidewalls are 5 µm thick (in-plane) and 40 µm high (equal to chamber depth), enabling nutrient exchange between perimeter channels and chamber interiors. A 700 µm-diameter epicentre port was punched for cell seeding. **(B)** Representative image of the internal HIC structure.

**Supplementary Figure S12. Uninfected controls distinguish infection-induced cell death from baseline cell loss in HIC. (A)** Representative dead-cell fluorescence image of an uninfected HIC after 7 days of culture. **(B)** Representative dead-cell fluorescence image of an HIC loaded with HCoV-229E-infected cells after 7 days of culture. These images are from the boxed lower-right region of HIC. Red = dead. Scale bar = 500 μm.

**Supplementary Figure S13. Effect of initial infection density (*I*_0_) on viral transmission in primary human lung fibroblasts. (A)** 96-well plates. Confocal images at days 0, 4, 7 for fixed *I*_0_ = 10³ and varying *S*_0_ = 10, 10³, 10⁵ (n = 3 wells per condition). Blue: initially infected cells (*I*_0_); green: susceptible cells (*S*_0_); red: dead cells. Cartoon icons indicate relative *I*_0_. **(B)** HIC spatial dynamics. Left: hexagonal-lattice maps at days 0, 4, 7 showing chamberwise counts of *S*_0_ (green scale) and *I*_0_ (red scale); dead cells indicated in dark red. Right: matching confocal images from the boxed lower-right region (n = 3 devices per condition). Blue: initially infected cells (*I*_0_); green: susceptible cells (*S*_0_); red: dead cells. **(C, D)** Quantification in **(C)** wells and **(D)** HIC. Time courses of remaining *S*_0_ over 7 days; *R*_0_ is estimated from the experimentally obtained *S*_0_-depletion curves.

**Supplementary Figure S14. Effect of imipramine treatment of initially infected cells on viral transmission in homogeneous wells and HIC. (A)** 96-well plates. Confocal images at days 0, 2, 4, and 7 for control, DMSO, and imipramine-treated groups at 0.25, 2.5, and 25 µM. Initially infected cells (*I*_0_) were suspended in the indicated drug-containing medium before loading, while susceptible cells (*S*_0_) were maintained in normal culture medium. Green: susceptible cells (*S*_0_); red: dead cells. Blue dashed boxes indicate enlarged regions. **(B)** HIC spatial dynamics. Hexagonal-lattice maps and corresponding confocal images at days 0, 2, 4, and 7 for control, DMSO, and imipramine-treated *I*_0_ groups. The pre-seeded *S*_0_ population was maintained in normal medium, and only *I*_0_ cells were suspended in imipramine-containing medium before loading into the epicentre. Left: lattice maps showing chamberwise counts of *S*_0_ and *I*_0_; dead cells are indicated in dark red. Right: matching confocal images from the boxed lower-right region. Green: susceptible cells (*S*_0_); red: dead cells. **(C, D)** Quantification in **(C)** 96-well plates and **(D)** HIC. Time courses of remaining *S*_0_ over 7 days; *R*_0_ is estimated from the experimentally obtained *S*_0_-depletion curves.

**References**

[1]	R. J. G. Hulswit, Y. Lang, M. J. G. Bakkers, et al., *Proceedings of the National Academy of Sciences of the United States of America* **2019**, *116* (7), 2681, <https://doi.org/doi:10.1073/pnas.1809667116>.

[2]	L. Dubois, N. Pietrancosta, A. Cabaye, et al., *Journal of Medicinal Chemistry* **2020**, *63* (15), 8231, <https://doi.org/10.1021/acs.jmedchem.9b02119>.

[3]	C. L. Yeager, R. A. Ashmun, R. K. Williams, et al., *Nature* **1992**, *357* (6377), 420, <https://doi.org/10.1038/357420a0>.
